# Optimised ARID1A immunohistochemistry is an accurate predictor of *ARID1A* mutational status in gynaecological cancers

**DOI:** 10.1002/cjp2.103

**Published:** 2018-07-20

**Authors:** Saira Khalique, Kalnisha Naidoo, Ayoma D Attygalle, Divya Kriplani, Frances Daley, Anne Lowe, James Campbell, Thomas Jones, Michael Hubank, Kerry Fenwick, Nicholas Matthews, Alistair G Rust, Christopher J Lord, Susana Banerjee, Rachael Natrajan

**Affiliations:** ^1^ The Breast Cancer Now Toby Robins Research Centre, Division of Breast Cancer The Institute of Cancer Research London UK; ^2^ Division of Molecular Pathology The Institute of Cancer Research London UK; ^3^ Gynaecology Unit The Royal Marsden NHS Foundation Trust London UK; ^4^ Department of Histopathology The Royal Marsden NHS Foundation Trust London UK; ^5^ ICR Core Bioinformatics Facility, The Institute of Cancer Research Sutton UK; ^6^ Molecular Diagnostics Department The Centre for Molecular Pathology, The Royal Marsden NHS Foundation Trust Sutton UK; ^7^ Tumour Profiling Unit The Institute of Cancer Research London UK; ^8^ The CRUK Gene Function Laboratory The Institute of Cancer Research London UK; ^9^ Division of Clinical Studies The Institute of Cancer Research London UK

**Keywords:** ARID1A immunohistochemistry, clear cell ovarian cancer, next generation sequencing, biomarker

## Abstract

*ARID1A* is a tumour suppressor gene that is frequently mutated in clear cell and endometrioid carcinomas of the ovary and endometrium and is an important clinical biomarker for novel treatment approaches for patients with *ARID1A* defects. However, the accuracy of ARID1A immunohistochemistry (IHC) as a surrogate for mutation status has not fully been established for patient stratification in clinical trials. Here we tested whether ARID1A IHC could reliably predict *ARID1A* mutations identified by next‐generation sequencing. Three commercially available antibodies – EPR13501 (Abcam), D2A8U (Cell Signaling), and HPA005456 (Sigma) – were optimised for IHC using cell line models and human tissue, and screened across a cohort of 45 gynaecological tumours. IHC was scored independently by three pathologists using an immunoreactive score. *ARID1A* mutation status was assessed using two independent sequencing platforms and the concordance between *ARID1A* mutation and protein expression was evaluated using Receiver Operating Characteristic statistics. Overall, 21 *ARID1A* mutations were identified in 14/43 assessable tumours (33%), the majority of which were predicted to be deleterious. Mutations were identified in 6/17 (35%) ovarian clear cell carcinomas, 5/8 (63%) ovarian endometrioid carcinomas, 2/5 (40%) endometrial carcinomas, and 1/7 (14%) carcinosarcomas. ROC analysis identified greater than 95% concordance between mutation status and IHC using a modified immunoreactive score for all three antibodies allowing a definitive cut‐point for *ARID1A* mutant status to be calculated. Comprehensive assessment of concordance of ARID1A IHC and mutation status identified EPR13501 as an optimal antibody, with 100% concordance between *ARID1A* mutation status and protein expression, across different gynaecological histological subtypes. It delivered the best inter‐rater agreement between all pathologists, as well as a clear cost‐benefit advantage. This could allow patients to be accurately stratified based on their ARID1A IHC status into early phase clinical trials.

## Introduction

Recent studies have highlighted that somatic loss of function mutations in the tumour suppressor gene *ARID1A* underpin 57% of ovarian clear cell carcinomas (OCCC) 1, 2, 40% of uterine endometrioid carcinomas 3 and between 20 and 36% of uterine carcinosarcomas 4, 5, 6; however, they are rare in high‐grade serous ovarian carcinoma (HGSOC)^1^, ^2^. HGSOCs are underpinned by *TP53* mutations, where p53 immunohistochemistry (IHC) is routinely used in clinical practice to aid diagnosis 7. However, *TP53* mutations are uncommon in OCCC; these differences in driver mutational profiles highlight the distinct aetiology of the two diseases. Moreover, the presence of *ARID1A* missense mutations may not alter ARID1A protein expression, unlike *TP53* missense mutations. Loss or significant reduction of ARID1A protein expression is associated with heterozygous *ARID1A* mutations 1, 8, 9, suggesting a dominant‐negative tumour suppressor role (see 10 for a comprehensive review). ARID1A forms a key DNA binding subunit in the ATP‐dependent SWItch/Sucrose Non‐Fermentable (SWI/SNF) chromatin‐remodelling complex that modulates the winding of DNA around histone cores allowing access to the DNA to enable transcription, DNA repair, and replication 11, 12; loss of function of this protein leads to aberrant cell cycle and loss of proliferation control 13. Two genetically engineered mouse models (GEMM) have been created with *ARID1A* alterations that led to tumour formation with loss of ARID1A protein expression observed by IHC. The first was an ovarian endometrioid tumour with co‐existent *PTEN* loss 14 and, more recently, a GEMM harbouring both *ARID1A* loss and a *PIK3CA* (H1047R) mutation promoted ovarian clear‐cell tumourigenesis 15. *ARID1A* mutations are also detected at high frequencies in other solid tumours including 23% of pancreatic ductal adenocarcinoma, 23% of advanced urothelial carcinomas, 18% hepatocellular carcinomas, and 6% of metastatic castrate‐resistant prostate cancers 16, 17, 18, 19, 20.

Novel ways of treating patients with *ARID1A* mutations have focused largely on using synthetic‐lethal approaches. Bitler *et al* highlighted the potential of targeting the antagonistic activity between SWI/SNF and EZH2 methyltransferase with the EZH2 small molecule inhibitor GSK126, which triggered apoptosis in *ARID1A* mutated cells 21. This was mediated via upregulation of *PIK3IP1*, a direct target of EZH2, and selectivity was further enhanced upon inhibition of PI3K‐AKT signalling 21. Subsequent work by Bitler *et al* has shown that *ARID1A*‐mutated ovarian cancers are selectively dependent on HDAC6 activity, due to HDAC6 upregulation in *ARID1A* mutant cells that mechanistically inactivates the apoptosis‐promoting function of *TP53* due to deacetylation of histone lysine 120 [22]. The work showed that treating *ARID1A*‐mutated tumours with the small molecule HDAC6 inhibitor, ACY1215, showed a survival benefit *in vivo*. Additional approaches identified reliance on the *ARID1A* paralogue *ARID1B* in mutant cells 23. Our group has found *ARID1A* defective OCCC tumours can be targeted with the multikinase inhibitor dasatinib, mediated through addiction to the dasatinib target YES1 [9] and more recently a profound sensitivity to inhibition of the DNA repair kinase ATR, leading to premature mitotic entry, genomic instability, and apoptosis 24. Shen *et al* have additionally shown that loss of ARID1A leads to impaired G2‐M DNA damage checkpoint activation and repair of DNA double strand breaks (DSB), causing sensitivity to DSB inducing treatments such as the Poly (ADP‐ribose) polymerase (PARP) inhibitor talozaparib, both *in vitro* and *in vivo* 25. Together, these observations open up the possibility of assessing these therapeutic approaches in clinical trials, in particular for tumour types with high frequencies of *ARID1A* mutations, such as OCCC.

Currently there are no clinical trials recruiting patients that prospectively assess *ARID1A* mutational status. However, there are three early phase trials investigating *ARID1A* mutational status and response to therapy, the first of which allocates treatment to patients whose biopsies are sequenced as part of on‐going clinical sequencing programmes outside of the remit of the clinical trial 26. Patients with *PIK3CA*, *AKT*, or *ARID1A* mutations will receive olaparib with the AKT inhibitor AZD5363 [26]. A second is a randomised phase II study of nintedanib (an oral tyrosine kinase inhibitor targeting VEGF receptors 1–3, FGFR 1–3, and PDGFR α and β) compared to chemotherapy in patients with clear cell carcinoma of the ovary or endometrium, which will assess *ARID1A* mutational status retrospectively and correlate with outcome 27. A third trial, which is ongoing but not currently recruiting, will assess dasatinib in patients with recurrent or persistent ovarian, Fallopian tube, endometrial, or peritoneal carcinoma and will retrospectively compare ARID1A mutational and IHC status 28. These trials highlight that prospectively assessing *ARID1A* mutational status is potentially cost‐prohibitive and the turn‐around time can make it difficult for trial recruitment. Therefore, a surrogate biomarker of mutational status such as IHC is needed.

Despite the clinical importance of *ARID1A* mutations, rapid sequencing is not widely available. Hence, IHC would be the commonest method to infer mutational status, highlighted by the fact that a number of open clinical trials are performing retrospective *ARID1A* mutational assessment. Studies that have so far assessed the correlation between *ARID1A* mutational status and IHC have demonstrated a good concordance 1, 3, 29. However, the accuracy of IHC as a predictor of *ARID1A* mutation in ovarian carcinoma has not been precisely defined as there is no uniform scoring system or specific antibody that is recommended for clinical IHC use. In the largest concordance study to date, Wiegand *et al* assessed ARID1A in 182 gynaecological tumours, defining positive staining as definitive nuclear staining and negative as no immunoreactivity 1. There was a statistically significant correlation between the loss of ARID1A protein expression and *ARID1A* mutational status in both OCCC and endometrioid carcinomas 1. In total, 73% of OCCC (27/37 cases) with a known *ARID1A* mutation showed loss of ARID1A expression. However, 11% of OCCC (4/36 cases) with no *ARID1A* mutation also showed loss of ARID1A protein expression 1. Of note this study used the mouse clone 3H2 (Abgent, CA), which targets a region of 111 amino acids (aa 1216 to 1326) but is no longer commercially available. Patients with recurrent or metastatic disease who will be entering the growing number of clinical trials where a robust assessment of ARID1A protein expression in the tumour could be informative will require a test that is accurate and reproducible with a fast turn‐around time.

Here our aims were to develop ARID1A IHC as a surrogate predictive biomarker for diagnostic assessment of *ARID1A* mutational status in gynaecological tumours. In particular, we sought to compare the concordance of a number of commercially available antibodies using a standardised scoring system and identify the most optimal assay for clinical assessment of mutation status.

## Materials and methods

### Cell lines

Cell lines ES2 and TOV21G were obtained from the American Type Tissue Collection (ATCC). HCT116 isogenic ARID1A (Q456*/Q456*) and parental lines were purchased from Horizon Discovery (Cambridge, UK). These were developed by knock‐in of a premature stop codon (Q456*). Cell lines were cultured in a humidified 37 °C incubator with 5% CO_2._ Cell lines were tested to confirm no mycoplasma infection using Mycoalert^™^ Mycoplasma Detection Kit as per manufacturer's instructions (Lonza, Slough, UK). Cell line identity was confirmed with short tandem repeat typing using the Promega GenePrint^®^10 system (Promega, Southampton, UK). Cell pellets were formalin fixed and paraffin‐wax embedded (FFPE) for antibody optimisation.

### Clinical samples

All patients gave written consent for the use of material for research purposes and tissue samples were obtained with appropriate ethical approval under the Royal Marsden Hospital (RMH) NHS Foundation Trust study: CCR3705 “Analysis of tumour specimens for biomarkers in gynaecological cancers” (Table 1 and supplementary material, Table S1). All patient samples were reviewed at RMH and appropriate FFPE tissue blocks were selected from their histology reports. Haematoxylin and eosin (H&E) sections were reviewed by a pathologist (DK) to confirm appropriate tumour tissue and content. Five thick (8 μm) sections were cut for DNA extraction, with an additional H&E slide and three unstained sections for ARID1A IHC. Whole serial sections were cut from the same diagnostic block to minimise heterogeneity between analysis for the ARID1A IHC and next‐generation sequencing (NGS). If no germline blood sample was available, then non‐malignant FFPE blocks were obtained and sections cut for DNA extraction.

**Table 1:** Overview of patient characteristics in the study

Parameter	Total number (*n*)
Number of tumour samples	45
Diagnosis	
Carcinosarcoma (CS)	7
Endometrial clear cell (ECC)	1
Endometrioid adenocarcinoma of the endometrium (EAE)	3
De‐differentiated carcinoma of the endometrium (DCE)	1
Endometrioid ovarian carcinoma (ENOC)	8
Low‐grade serous ovarian carcinoma (LGSOC)	3
High‐grade serous ovarian carcinoma (HGSOC)	1
Small cell carcinoma of the ovary, hypercalcaemic type (SCCOHT)	2
Mesonephric adenocarcinoma of the ovary (MAO)	1
Mesonephric adenocarcinoma of the ovary and endometrium (MAOE)	1
Ovarian clear cell carcinoma (OCCC)	17
Grade	
I	6
II	9
III	30
FIGO stage	
I	14
II	14
III	13
IV	4
Median age, years (range)	57, (21–76)
Endometriosis	
Yes	16
No	29
Primary specimen	
Yes	42
No	3

Patients were aged between 21 and 76 years of age, and comprised of eight gynaecological subtypes, the most frequent being clear cell carcinoma of the ovary, *n* = 17.

### DNA extraction and library preparation

DNA extraction and NGS took place in Good Clinical Laboratory Practice (GCLP)‐accredited laboratories at The Centre for Molecular Pathology, The Royal Marsden NHS Foundation Trust, Sutton. Genomic DNA from FFPE tissue sections was extracted using QIAamp FFPE Tissue Kit (Qiagen, Manchester, UK) according to the manufacturer's instructions for both tumour and non‐malignant content. Genomic DNA from blood was extracted using the QIAamp Blood mini kit (manual) or QIAsymphony DNA Midi Kit (automated) (Qiagen) according to the manufacturer's instructions. DNA quality was assessed on the Agilent 2200 Tapestation (Agilent, Stockport, UK) and the Qubit Fluorometer (Fisher Scientific, Loughborough, UK).

### Targeted *ARID1A* sequencing

*ARID1A* mutations were identified using a targeted capture panel (Nimblegen, Roche, Welwyn Garden City, UK), designed to target 59 genes for the FOrMAT clinical trial (Feasibility of Molecular Characterization Approach to Treatment, CCR3994, Royal Marsden NHS Hospital, Foundation Trust). The panel typically covers *ARID1A* at 99% >250X (supplementary material, Table S2). Individual sample library preparation was performed using the KAPA Biosystem HyperPlus kit using 50–200 ng DNA. Pooling and capture steps were performed using the Nimblegen SeqCap Capture Protocol (Roche). KAPA Library Quant Kit Universal qPCR Mix (Illumina), was used to quantify the libraries before combining in equimolar concentrations prior to sequencing on the Illumina MiSeq. A high confidence call covered >95% of a region; medium confidence >85% and a failure if the coverage was <85% coverage. MiSeq Reporter software (v2.5.1) was used to align sequences to version hg19 of the human genome using aligner: BWA v0.61 and Somatic Caller v3.5.2.1 to call variants alongside in‐house web server 1.0 and 2.0 software. Variants were called at a frequency of 5% and present in at least 5 reads, with a minimum read depth of 10 reads. Mutations were manually visualized in the Integrated Genomics Viewer. All mutations were validated using a custom AmpliSeq panel (Thermo Fisher, Wilmington, USA) and run on the Ion Proton panel (Thermo Fisher) with 10 ng input DNA. Sequencing data were analysed using the Ion Torrent software suite (version 5.2.2). Sequences were aligned to version hg19 of the human genome and mutations were called using the variantCaller (version v5.2.0.34). For pairs of tumour‐normal samples, mutation calls were intersected using vcf‐isec (VCFtools version 0.1.10) and filtered to retain somatic mutations. Oncotator and SnpEff (version 3.3h) were used to annotate somatic variants reporting the most deleterious effect. Further annotations were added to each mutation using ANNOVAR and the following databases: ClinVar, COSMIC, dbnsfp33a, clinvar_20170130, ljb23_ma, exac03, and exac03nontcga. Only those mutations that were confirmed with both sequencing platforms were included in the final concordance analysis. Raw targeted sequencing data have been deposited into the NCBI Sequence Read Archive under the accession PRJNA432413 and PRJNA432343.

**Table 2:** Summary of averaged pathologist IHC scores and validated sequencing results for ARID1A

Patient identifier	Diagnosis	IHC ARID1A antibody EPR13501 average score (Pathologists 1,2, and 3)	IHC ARID1A antibody D2A8U average score (Pathologists 1,2, and 3)	IHC ARID1A antibody HPA005456 average score (Pathologists 1,2, and 3)	IHC: ARID1A protein loss	Sequencing: *ARID1A* mutation and protein change
3705‐0442	CS	12	12	10	No	No
3705‐0449	CS	12	11	10	No	No
3705‐0456	CS	10	11	9	No	No
3705‐0470	CS	12	8	10	No	No
3705‐0484	CS	12	12	12	No	No
3705‐0500	CS	5	5	4	Yes	p.Tyr1377Ter, p.Glu1542Ter
3705‐0510	CS	12	12	8	No	No
666179	ECC	12	11	8	No	No
3705‐0142	EAO	7	1	4	Yes	p.Pro7fs
3705‐0341	EAO	12	11	9	No	No
3705‐0481	EAO	0	0	2	Yes	p.Pro1135fs
3705‐0541	EAO	NA	NA	NA	NA	p.Gln428Ter
3705‐0323	EAO	3	3	6	Yes	p.Gln512Ter, p.Arg1899Ter
3705‐0460	EAO	1	1	3	Yes	p.Arg1505Ter
3705‐0482	EAO	12	12	12	No	No
3705‐0529	EAO	12	12	9	No	No
3705‐0199	EAE	3	2	1	Yes	p.Ile1975fs, p.Gln799fs
3705‐0438	EAE	1	1	1	Yes	p.Arg1504Ter
3705‐0548	EAE	12	11	12	No	No
3705‐0553	DCE	9	6	7	No	No
3705‐0475	HGSOC	12	12	12	No	No
3705‐0487	LGSOC	11	12	11	No	No
3705‐0493	LGSOC	12	12	12	No	No
3705‐0525	LGSOC	11	11	6	No	No
3705‐0051	MAOE	12	11	12	No	NA failed sequencing QC
3705‐0308	MAO	12	12	7	No	No
3705‐0145	OCCC	0	3	2	Yes	p.Ser614Ter
3705‐0207	OCCC	12	10	8	No	No
3705‐0346	OCCC	2	1	3	Yes	p.Ala42fs, p.GLn723Ter
3705‐0379	OCCC	12	11	12	No	No
3705‐0383	OCCC	12	11	11	No	No
3705‐0416	OCCC	0	1	2	Yes	p.Gly191fs, p.Tyr485Ter
3705‐0435	OCCC	11	12	8	No	No
3705‐0453	OCCC	12	12	9	No	No
3705‐0464	OCCC	1	1	1	Yes	p.Gly1848fs, p.Ile2275Ser, p.Gln2070Ter
3705‐0468	OCCC	12	11	12	No	No
3705‐0497	OCCC	12	12	12	No	No
3705‐0514	OCCC	12	11	12	No	No
3705‐0540	OCCC	1	0	1	Yes	p.Gln932fs
3705‐0544	OCCC	12	12	12	No	No
3705‐0545	OCCC	0	0	3	Yes	p.Gly319fs, p.Leu1100fs
3705‐0558	OCCC	11	11	11	No	No
3705‐0435*	OCCC	12	12	12	No	No
3705‐0466	SCCOHT	12	12	12	No	No
3705‐0551	SCCOHT	12	12	12	No	No

CS, carcinosarcoma of the ovary; DCE, de‐differentiated carcinoma of the endometrium; ECC, endometrial clear cell; EAE, endometrioid adenocarcinoma of the endometrium; EAO, endometrioid adenocarcinoma of the ovary (encompassed within ENOC, endometrioid ovarian carcinoma); LGSOC, low grade serous ovarian carcinoma; HGSOC, high grade serous ovarian carcinoma; SCCOHT, small cell carcinoma of the ovary, hypercalcaemic type; MAOE, mesonephric adenocarcinoma of the ovary and endometrium; MAO, mesonephric adenocarcinoma of the ovary; NA, not available; OCCC, ovarian clear cell carcinoma.

Matched patient lung metastasis.

### ARID1A immunohistochemistry

IHC was performed on 3–4 μm thick whole tissue sections. The slides were incubated with antibodies to: Anti‐ARID1A, rabbit monoclonal 1:1000, EPR13501 (Abcam, Cambridge, UK), ARID1A/BAF250A, rabbit monoclonal 1:250, D2A8U (Cell Signaling Technology Europe, Leiden, The Netherlands), and anti‐ARID1A, rabbit polyclonal 1:400, HPA005456 (Sigma‐Aldrich, Gillingham, Dorset, UK), using the Dako‐Autostainer Link 48 with the EnVision FLEX kit as per manufacturer's instructions (Agilent Technologies, Cheadle, Cheshire, UK). Human breast, prostate, and kidney tissues were used as positive controls and xenograft models were obtained as previously described 24.

Cases were independently scored using an immunoreactive scoring system 30 by three pathologists, DK (Pathologist 1) KN (Pathologist 2), and AA (Pathologist 3), who were blinded to the sequencing results. Sections were evaluated for both intensity (0= negative, 1= weak staining; 2= moderate; 3= strong) and proportion of positively stained cells expressed as a percentage (0 = 0%; 1+ ≤10%; 2+= 11–50%; 3+= 51–80%; 4+>80%). The intensity and proportion of stained cells were multiplied to produce the final score between 0 and 12 [30]. Stromal cells were used as an internal positive control. The pathologists' scores were averaged to give a combined score used in further analyses. An IHC score “cut‐off” for loss of expression was defined in conjunction with the genomic deleterious mutation results for each antibody using receiver operating characteristic (ROC) statistical analysis. Fleiss kappa statistics were used to assess interrater variability 31.

## Results

### ARID1A IHC optimisation shows nuclear immunoreactivity in *ARID1A* wild‐type cases and absence in *ARID1A* mutant cases

**Figure 1:**
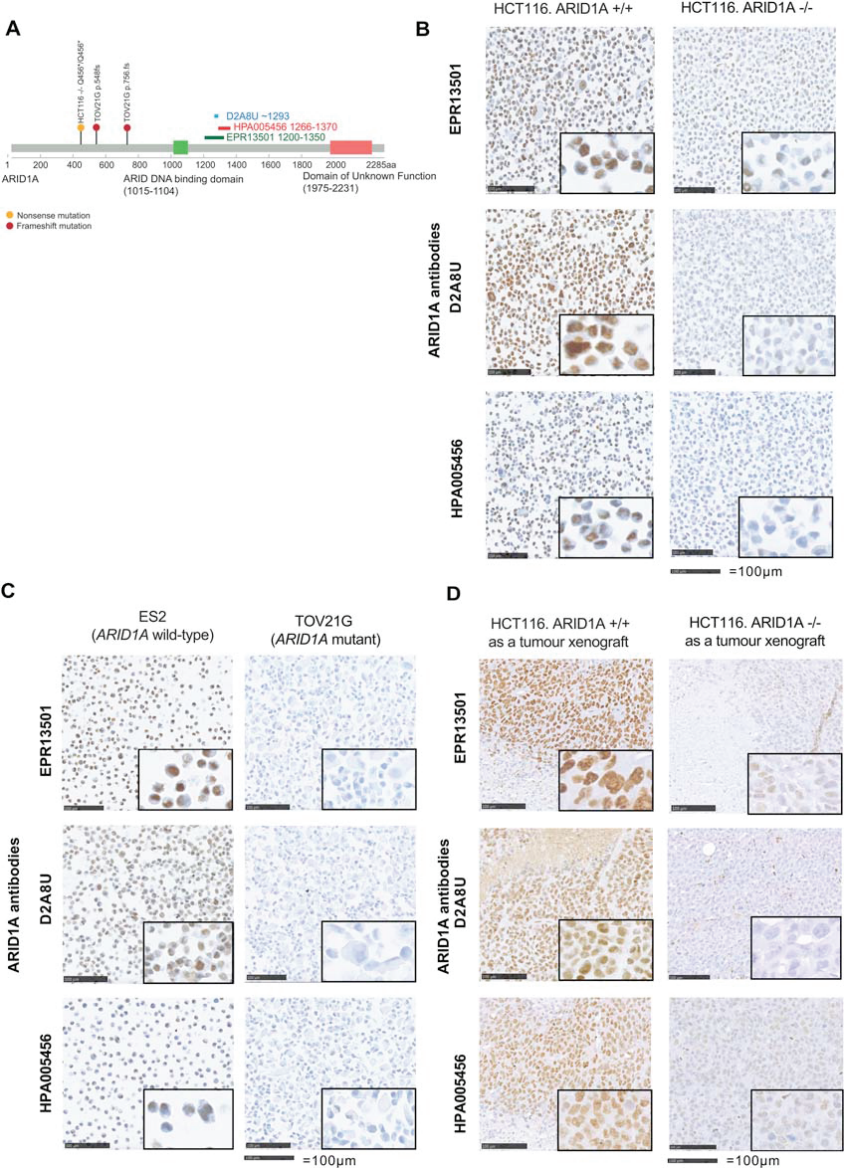
ARID1A antibody optimisation in tumour cell lines and tumour xenograft models. (A) Schematic of the ARID1A protein illustrating epitope regions of all three antibodies studied. The lollipop plot shows mutation loci of two cell lines used in antibody optimisation: HCT116 *ARID1A* isogenic mutant cell line Q456*/Q456*, nonsense mutation (depicted in orange); and TOV21G, a compound heterozygous cell line with mutation loci TOV21G p.548fs and p.756fs, frameshift mutations (depicted in mauve). (B) Immunoreactivity of ARID1A detected by all three antibodies in HCT116 *ARID1A* isogenic cell lines embedded in FFPE blocks. HCT116 –/– (*ARID1A* mutant) shows loss of ARID1A immunoreactivity whereas nuclear immunoreactivity was preserved in the HCT116 +/+ (*ARID1A* wild‐type) cell line. EPR13501, rabbit monoclonal, antigen retrieval using microwave and dilution 1:1000. D2A8U, rabbit monoclonal, antigen retrieval with pretreatment module and dilution 1:250. HPA005456, rabbit polyclonal, antigen retrieval with pretreatment module and dilution 1:400. Scale bar is equal to 100 µm. (C) ARID1A immunoreactivity in ovarian clear cell carcinoma cell lines. TOV21G (*ARID1A* mutant) shows loss of ARID1A immunoreactivity whereas immunoreactivity was preserved in the ES2 (*ARID1A* wild‐type) cell line. Scale bar is equal to 100 µm. (D) ARID1A immunoreactivity was detected with all three antibodies in xenograft models of HCT116 +/+ with loss of ARID1A expression in HCT116 –/– xenograft models. The background staining seen in cell lines was reduced in xenografts. Scale bar is equal to 100 µm.

We first evaluated three ARID1A antibodies based upon their current availability and recent use in the literature 24, 32, 33; Abcam EPR13501 monoclonal antibody, Cell Signaling D2A8U monoclonal antibody, and Sigma HPA005456 polyclonal antibody, hereafter abbreviated to clone details (Figure 1A). Antibodies were optimised on human tissue, HCT116 *ARID1A* isogenic (mutant and wild‐type) cell lines, ES2 (*ARID1A* wild‐type), TOV21G (*ARID1A* mutant) ovarian clear cell carcinoma cell lines, and in HCT116 *ARID1A* isogenic xenograft models (Figure 1B‐D). These cell lines are known to either express or not express ARID1A as previously evaluated by western blot (supplementary material, Figure S1 and 24). Antibodies were diluted accordingly to ensure a contrast between mutant and wild‐type cell lines. ARID1A immunoreactivity was detected in the nucleus, in both malignant epithelial tumour and stromal cells, which were used as a positive internal control in all samples. Of all three antibodies tested, D2A8U showed the strongest immunoreactivity in the cell line models (Figure 1C), with background staining most visible with HPA005456.

**Figure 2:**
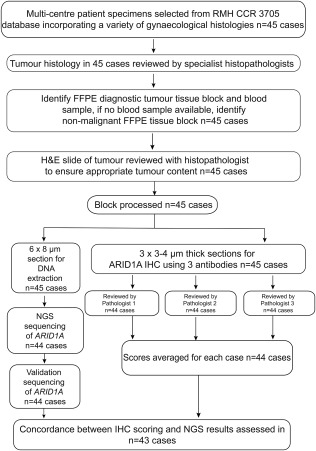
Study workflow. Modified CONSORT diagram showing the processing of the 45 gynaecological cancer cases identified. After histopathology review, a representative block was chosen. The same FFPE block was used for extracting DNA for next‐generation sequencing (NGS) and IHC. One case failed NGS quality control and one case was not suitable for IHC analysis. Three histopathologists independently reviewed and scored cases. These were then averaged and compared to mutational status to establish concordance (*n* = 43).

We next evaluated ARID1A protein expression in a cohort of 45 gynaecological cancers with all three antibodies (Figure 2 and Table 1). All three antibodies demonstrated ARID1A immunoreactivity and performed well on archival tissue, with the oldest block evaluated from 2005 (3705‐0481) and the most recent from 2016 (666179) (Table 2 and supplementary material, Table S3). One case (3705‐0541) was not fixed appropriately at the time of resection and we were unable to process it for IHC, although we were able to extract good quality DNA. Twenty‐four cases scored a maximum immunoreactive score of 12 with EPR13501, compared to 16 with D2A8U and 13 with HPA005456. Four cases scored 0 with EPR13501, three cases scored 0 with D2A8U and none scored 0 with HPA005456 (Table 2). The scoring concordance of the antibodies between the pathologists varied, with EPR13501 showing the best inter‐rater agreement of 0.78, followed by D2A8U (0.67) and HPA005456 (0.57), Fleiss' kappa statistics 31 (supplementary material, Table S4).

**Table 3:** ROC curve analysis to define mutant immunoreactive score and concordance

Antibody	All gynaecological cases: ARID1A mutant score	Concordance (%)	OCCC cases: ARID1A mutant score	Concordance (%)
EPR13501	<8	100	<6.5	100
	100% sensitivity and 100% specificity		100% sensitivity and 100% specificity	
D2A8U	<5.5	100	<6.5	100
	100% sensitivity and 100% specificity		100% sensitivity and 100% specificity	
HPA005456	<6.5	97	<5.5	100
	100% sensitivity and 96% specificity		100% sensitivity and 100% specificity	

### Endometriosis‐related carcinomas show enrichment of *ARID1A* mutations

**Figure 3:**
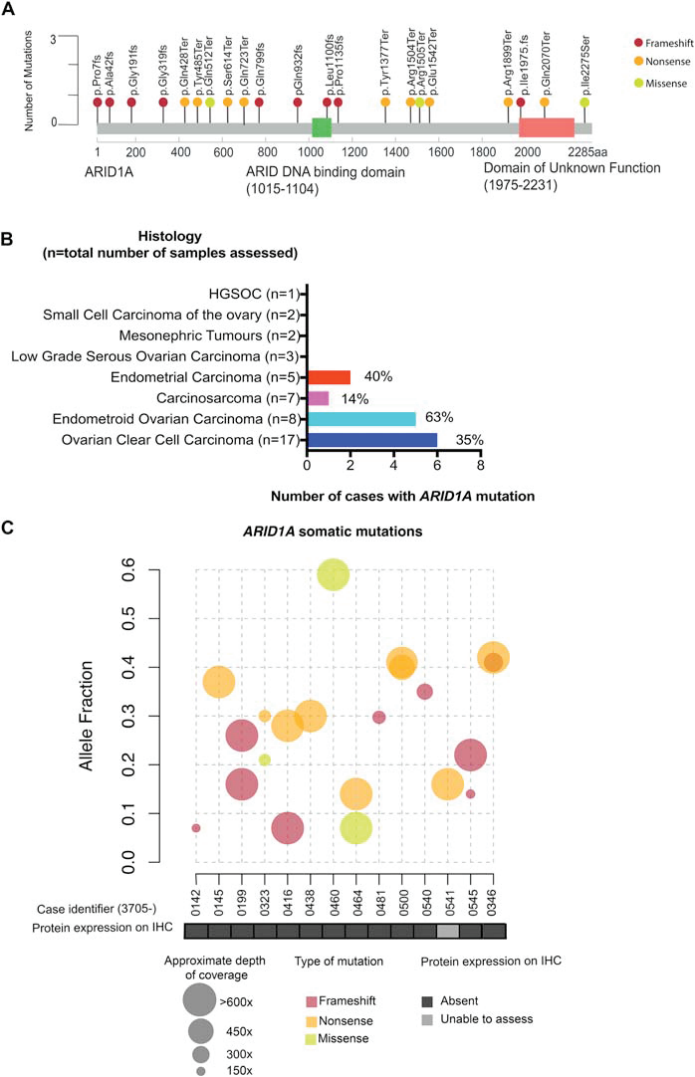
Distribution of *ARID1A* mutations identified by targeted sequencing in gynaecological cancers. (A) A lollipop plot showing the distribution and classes of validated loss of function mutations in *ARID1A* detected by both sequencing platforms in 44 cases. Twenty‐one *ARID1A* mutations were identified in 14 patients. The majority were frameshift mutations (9, in mauve), with 11 nonsense mutations (orange) and 1 missense mutation (green). (B) Bar chart showing the frequency of *ARID1A* mutations according to histology. An enrichment of *ARID1A* mutations was seen in endometriosis‐related tumours and carcinosarcomas, with 63% of endometroid ovarian carcinomas (light blue), and 35% of OCCCs (dark blue) having an *ARID1A* mutation. Endometrial carcinoma includes ECC, EAE and DCE. (C) A VAF plot shows each case with the type of validated mutation (colour of circle) and coverage (size of circle) with corresponding protein expression on IHC (boxes below). Six cases had more than one mutation in *ARID1A*. Case 3705‐0541 was not suitable for IHC processing.

*ARID1A* mutations were next evaluated using a 59‐gene targeted DNA capture panel, followed by NGS with a median depth of 699X for *ARID1A*, (mean depth range of *ARID1A* 117X‐2711X) (Figure 2 and supplementary material, Table S2). We were able to extract and sequence good quality DNA from archival FFPE blocks in 44 out of 45 cases (exception: 3705‐0051). Twenty‐five *ARID1A* mutations were identified in 16 cases, of which 21 mutations were validated in 14 cases, and were spread throughout the gene, consistent with previous studies 1, 2 (Figure 3A and supplementary material, Table S5). The frequencies of mutations according to histological subtype were 6/17 (35%) in OCCC, 5/8 (63%) in endometrioid adenocarcinoma of the ovary, 17% (1/6, a pelvic carcinosarcoma case) in carcinosarcomas, and 40% in endometrial carcinoma (2/5, both endometrioid endometrial carcinomas) (Table 2 and Figure 3B), in keeping with reported frequencies in the literature 2, 6, 34. We identified 9 frameshift mutations, 11 nonsense mutations, and 1 missense mutation (Table 2 and Figure 3A,C,D). Six cases had more than one mutation (Table 2 and Figure 3C). The variant allele frequency (VAF) ranged from 0.07 to 0.59, with a number of the mutations showing a low VAF, suggesting subclonal tumour populations (e.g., case 3705‐0464 with two mutations, with VAFs of 0.07 and 0.14). Of note, protein loss occurred even with a low VAF, for example in a carcinosarcoma case, 3705‐0481, with a frame shift mutation (p.Pro1135fs) with a VAF of 0.19, scores of 0 (EPR13501), 0 (D2A8U), and 2 (HPA005456) (Figure 3C). Two cases (3705‐0553, 666179) had mutations that were not validated on the Ion Torrent (supplementary material, Table S5) due to coverage issues.

### ARID1A immunoreactivity and mutational status shows between 97 and 100% concordance in gynaecological cancers

**Figure 4:**
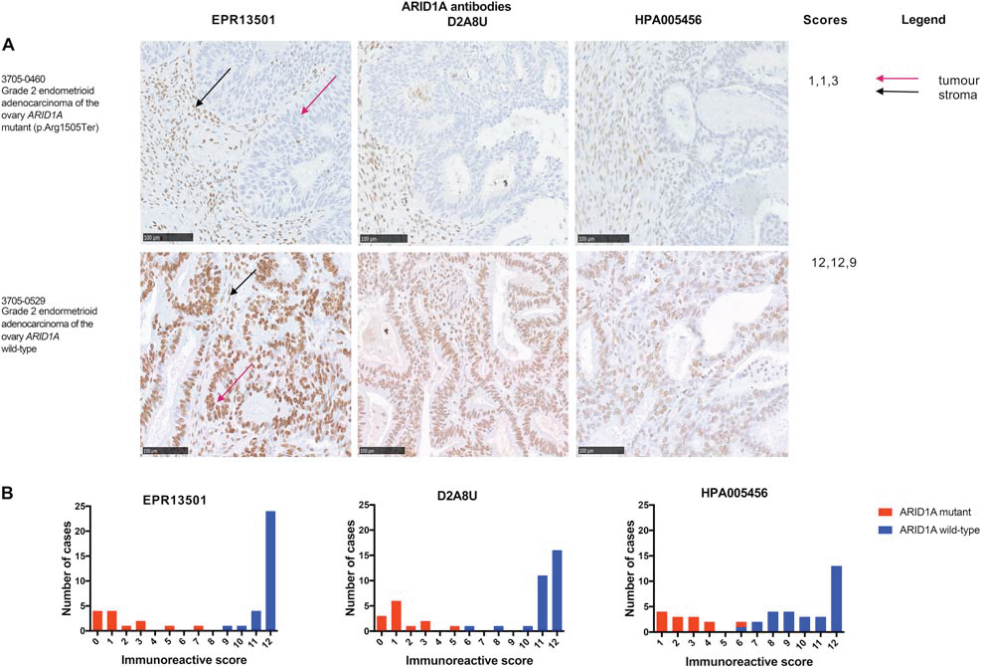
ARIDIA IHC shows good concordance with mutational analysis for all three antibodies in a variety of gynaecological cancers. (A) ARID1A immunoreactivity in grade 2 endometrioid adenocarcinoma of the ovary. Case 3705‐0460, with one mutation (p.Arg1505Ter), shows a lack of tumour cell staining with positive stromal staining (immunoreactive score with EPR13501: 1, D2A8U: 1 and HPA005456: 3). Case 3705‐0529, *ARID1A* wild‐type, shows positive tumour cell nuclear staining with an immunoreactive score with EPR13501: 12, D2A8U: 12 and HPA005456: 9. Scale bar is equal to 100 µm. (B) Histograms showing the distribution of all immunoreactive scores (*n* = 43) with all three antibodies, annotated with validated mutational status. The majority of *ARID1A* mutant cases (red) show low immunoreactivity scores and *ARID1A* wild‐type cases (blue) show high immunoreactivity scores. There is greatest variation in the HPA005456 scores.

**Figure 5:**
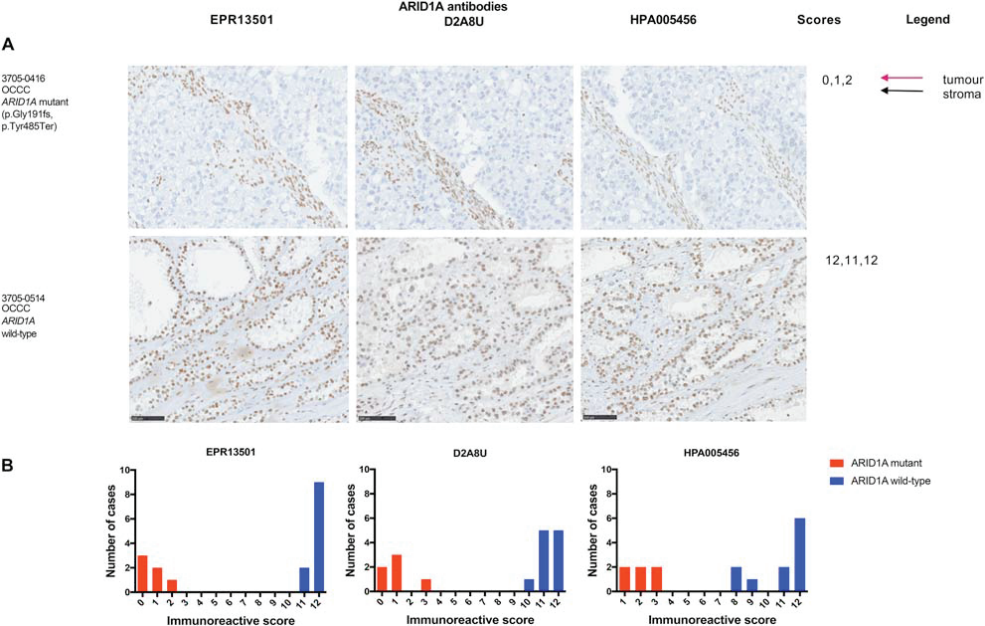
ARIDIA IHC shows good concordance with mutational analysis for all three antibodies in OCCC. ARID1A immunoreactivity in OCCC. Case 3705‐0416, with two mutations (p.Gly191fs, p.Tyr485Ter), shows a lack of tumour cell staining with positive stromal staining (immunoreactive score with EPR13501: 0, D2A8U: 1 and HPA005456: 2). Case 3705‐0514, *ARID1A* wild‐type, shows positive tumour cell nuclear staining with an immunoreactive score with EPR13501: 12, D2A8U: 11, and HPA005456: 12. Scale bar is equal to 100 µm. (B) Histogram showing immunoreactivity scores for OCCC cases shows a bimodal distribution between mutant cases (loss of immunoreactivity) and *ARID1A* wild‐type cases (retention of immunoreactivity).

In order to assess if IHC could be used as a biomarker of mutation status, we next evaluated the concordance of ARID1A IHC with the validated mutations. Using ROC curve analysis, we were able to define a cut‐off for each antibody to reliably identify mutant cases (Table 3). Concordance between mutated gynaecological carcinoma cases and the averaged IHC scores (supplementary Table S3) were calculated, including a specific analysis for OCCC cases (Table 3). One of the six carcinosarcomas, 3705‐0500, was an outlier as, although it had two nonsense mutations (pTyr1377Ter, p.Glu1542Ter) at relatively high VAF's (0.4), there was only a slight reduction in protein expression, scoring 5 (EPR13501), 5 (D2A8U), and 4 (HPA005456). Both subtypes of serous ovarian carcinoma, a mesonephric adenocarcinoma case and the small cell carcinomas of the ovary, hypercalcaemic type, were *ARID1A* wild‐type and showed high protein expression (with scores ranging between 11 and 12 (EPR13501), 11 and 12 (D2A8U) and 6 and 12 (HPA005456) (Figure 4). Of all three antibodies tested, HPA005456 showed the widest range of scores (Figure 4B). Seven OCCC cases were found to have *ARID1A* mutations, all of which showed a reduction in immunoreactivity (Figure 5). All OCCC *ARID1A* wild‐type cases had detectable protein expression, with scores between 11 and 12 with EPR13501.

## Discussion

In this study, we assessed the concordance of three commercially available antibodies for the assessment of ARID1A protein expression as a surrogate biomarker of mutation status. Overall, we found that IHC is an excellent surrogate biomarker of loss of function mutational status and were able to establish thresholds with each antibody to reliably identify mutant cases to be used in prospective patient assessment that improves upon published concordance rates.

Concordance rates for all cases were 100% (EPR13501), 100% D2A8U, and 97% (HPA005456). The histograms for all antibodies show that overall there is a clear bimodal distribution in immunoreactive scores between the mutant and wild‐type cases. This is best exemplified with EPR13501 and D2A8U, with HPA005456 showing the greatest variation, perhaps due to the polyclonal nature of the antibody, whereas the D2A8U and EPR13501 are monoclonal. For instance, comparing OCCC cases alone, our concordance was 100% with all antibodies, compared to 73% (27/37 samples) as reported by Wiegand *et al* 1. Given that the number of OCCC mutant cases, we assessed is small due to the rarity of cases and the fact that trials combine gynaecological histologies, we propose utilising the cut‐offs derived from all gynaecological cases. As all three antibodies had excellent specificity and sensitivity, other factors come into consideration when deciding which antibody to recommend taking forward for potential clinical use. The interpathologist scores were most consistent with EPR13501, and the dilution factor of 1:1000 for EPR13501 compared to D2A8U's 1:250 means that this would be more cost‐effective with less batch‐to‐batch variation. D2A8U's staining was most intense in cell line models, but weaker in human tissue, whereas EPR13501 had the strongest intensity in human tissue out of all three antibodies. We investigated HPA005456 as it had been used in a number of recent papers 3, 33, 35; however, as it is a rabbit polyclonal antibody, it will not be available for investigators once the stock runs out and is therefore not a long term viable option.

One of the limitations of this study is that even though we identified 45 patient samples initially, we were only able to assess 43 cases. However, the rarity of these tumours in general renders larger series challenging. In order to correct for this, we were stringent with our analysis of the sequencing data, including only cases where mutations were identified by both sequencing platforms. Thus, we could be confident with the mutation calls that we made. Overall, we have shown that IHC is generally concordant with mutational status; we did observe some variation in the carcinosarcoma cases, which may, in itself, reflect the heterogeneity of these tumours and the limitations of using an immunoreactive score. For example, in case 3705‐0500, this may be explained by the *ARID1A* mutation occurring after the ARID1A epitope sites and hence the detection of the truncated residual protein. We observed one endometrioid ovarian adenocarcinoma with a small area of subclonal loss (supplementary material, Figure S2). The patient did not have an identifiable *ARID1A* mutation and their tumour had an ARID1A IHC score of 12 (>80% of tumour staining strongly). How such patients respond to targeted therapy will only be evaluable in the context of a clinical trial. Of note, we did not identify any somatic in‐frame deletions. However, given reports that such mutations affect the subcellular distribution of ARID1A 36, it would be interesting to assess this with the optimal antibody.

Although a number of smaller studies have assessed concordance between ARID1A IHC and mutational status in gynaecological carcinomas, the concordance rates are lower than we report here and the antibody details and scoring systems used are not fully reported 29, 31, 32, 33. For instance, Lheureux *et al* analysed archival tissue by IHC and mutational status as part of a 40‐patient phase II clinical trial in OCCC with ENMD‐2076, an oral multitargeted kinase inhibitor 37. They had paired data for 32 samples, with 19 *ARID1A* mutant cases and 13 wild‐type cases, where the concordance of the mutational status with IHC was only 69%. Furthermore, the antibody and specific scoring details and nature of mutations were not detailed. Guan *et al* compared ARID1A IHC and mutational status in uterine carcinomas using the polyclonal HPA005456 antibody (targeting amino acids 1266–1370), with negativity defined as absence of nuclear staining, and identified that only 50% (5/10) of cases with deleterious mutations showed complete lack of ARID1A expression. Our study only included gynaecological tumours and would need to be extended to other tumour types to ascertain its general applicability.

A recent sequencing study identified cancer‐associated inactivating *ARID1A* mutations in deep infiltrating endometriosis, with loss of ARID1A immunoreactivity serving as a surrogate for *ARID1A* inactivating mutations using the HPA005456 antibody 35. Using the monoclonal EPR13501 antibody, which had the best inter‐rater agreement in our study, would allow comprehensive analysis of endometriosis to determine the clinical significance of ARID1A loss and be of potential diagnostic use.

In conclusion, we have systematically assessed a number of commercially available antibodies and identified EPR13501 as a robust biomarker of *ARID1A* status with a cut‐off of <8 using our optimised scoring system. This will be useful for recruiting patients for clinical trials based on *ARID1A* mutational status. An international academic trial of ATR inhibition in combination with a PARP inhibitor in ARID1A‐stratified gynaecological cancers that utilises our findings is planned to open in 2018 using this approach, allowing validation and evaluation of the IHC scoring system in the context of a prospective clinical trial.

## Author contributions statement

RN, CJL, and SB conceived and designed the study. SK, KN, AA, DK, FD, AL, TJ, MH, KF, and NM carried out the experiments and undertook IHC scoring. JC, TJ, MH, AGR, and RN performed the bioinformatics analysis. SK, KN, AA, DK, FD, CJL, and SB discussed and interpreted the results. SK, CJL, SB, and RN wrote the first draft. All authors read and approved the final manuscript.

## Supporting information

SUPPLEMENTARY MATERIAL ONLINE

**Figure S1.** ARID1A status of OCCC cell lines. Western blot of ARID1A protein expression in ES2 (OCCC *ARID1A* wild‐type cell line) and TOV21G (OCCC *ARID1A* mutant cell line) using the EPR13501, D2A8U, and HPA005456 antibodies. ARID1A (250 kDa fragment), loading control β‐Actin (42 kDa)

**Figure S2.** Subclonal expression in a Grade 2 endometrioid adenocarcinoma of the ovary (case 3705‐0482). Images of an area of subclonal expression with IHC scores and sequencing results. Top Row: Low power magnification, arrow shows area of absent protein expression surrounded by area of positive expression with all three antibodies (scale bar 1 mm). Bottom Row: High power magnification, arrow shows area of absent protein expression surrounded by area of positive expression with all three antibodies (scale bar 100 µm)

**Table S1.** Extended table of clinicopathological features of the patient cohort

**Table S2.** Summary of *ARID1A* sequencing metrics from the targeted capture panel

**Table S3.** Summary of individual and combined scores for ARID1A IHC

**Table S4.** Inter‐pathologist concordance metrics

**Table S5.** Summary of *ARID1A* mutations identified and their validation
