# Supplementary figures and images for: Optimised ARID1A immunohistochemistry is an accurate predictor of ARID1A mutational status in gynaecological cancers
Source: J Pathol Clin Res. 2018 Jul 20;4(3):154–66. doi: 10.1002/cjp2.103 (PMC6065117; doi:10.1002/cjp2.103)

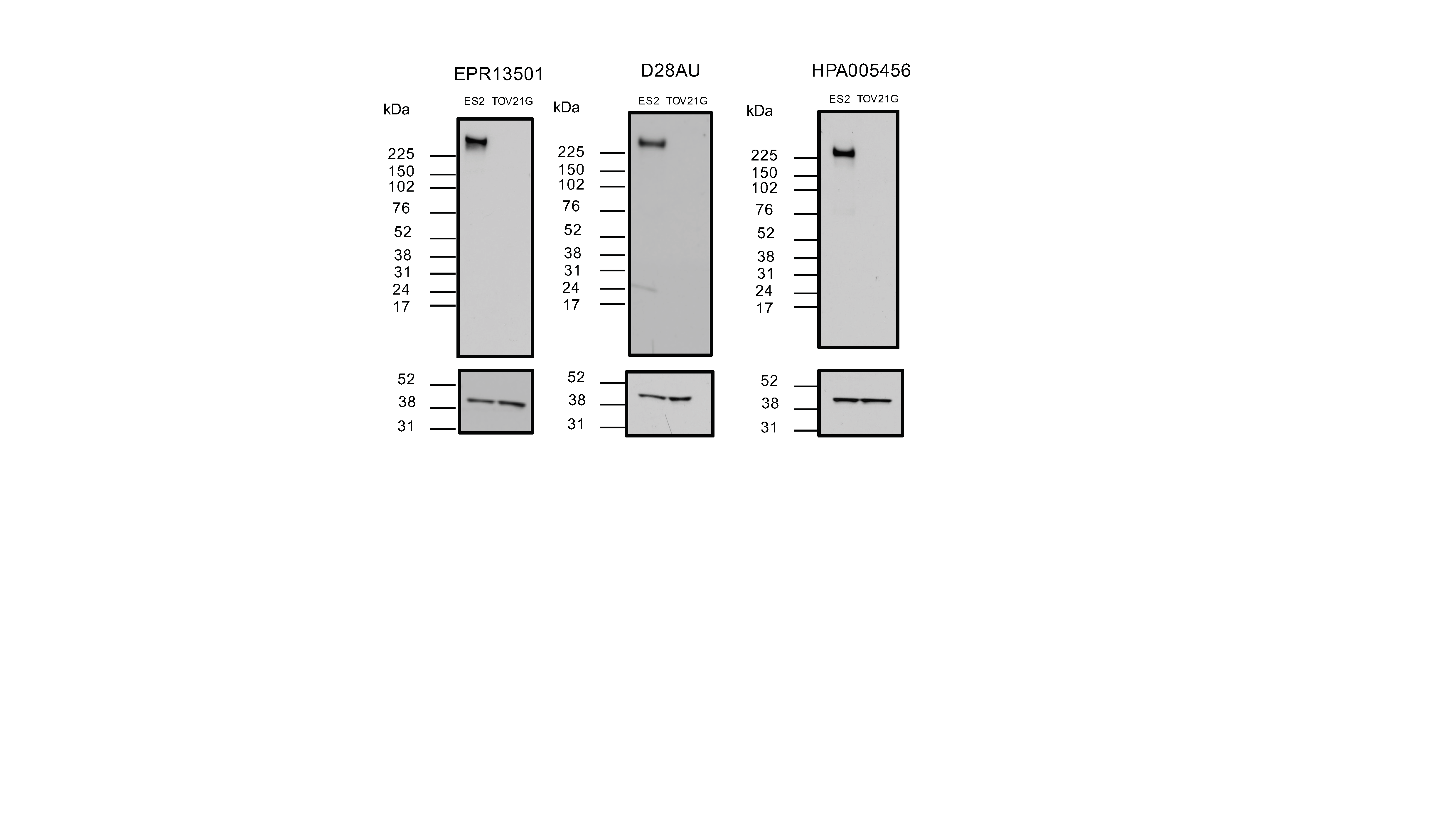

Supplement: Supplementary file 1 — Figure S1. ARID1A status of OCCC cell lines. Western blot of ARID1A protein expression in ES2 (OCCC ARID1A wild‐type cell line) and TOV21G (OCCC ARID1A mutant cell line) using the EPR13501, D2A8U, and HPA005456 antibodies. ARID1A (250 kDa fragment), loading control β‐Actin (42 kDa) [file CJP2-4-154-s001.tiff]

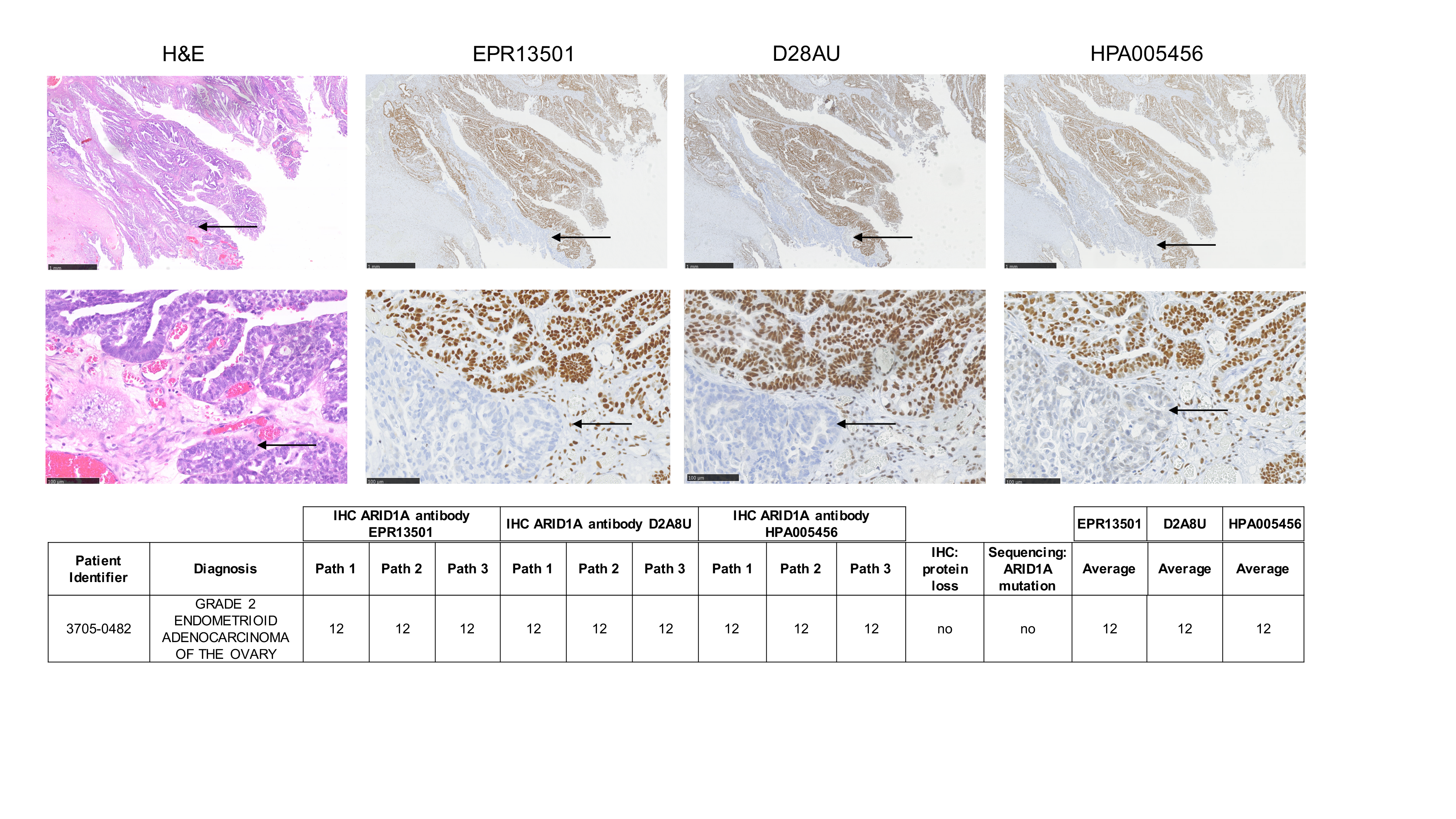

Supplement: Supplementary file 2 — Figure S2. Subclonal expression in a Grade 2 endometrioid adenocarcinoma of the ovary (case 3705‐0482). Images of an area of subclonal expression with IHC scores and sequencing results. Top Row: Low power magnification, arrow shows area of absent protein expression surrounded by area of positive expression with all three antibodies (scale bar 1 mm). Bottom Row: High power magnification, arrow shows area of absent protein expression surrounded by area of positive expression with all three antibodies (scale bar 100 µm) [file CJP2-4-154-s002.tiff]
