# Supplementary material for: Optimised ARID1A immunohistochemistry is an accurate predictor of ARID1A mutational status in gynaecological cancers
Source: J Pathol Clin Res. 2018 Jul 20;4(3):154–66. doi: 10.1002/cjp2.103 (PMC6065117; doi:10.1002/cjp2.103)
Supplement: Supplementary file 3 — Table S1. Extended table of clinicopathological features of the patient cohort [file CJP2-4-154-s003.docx]

**Table S1.** Extended table of clinicopathological features of the patient cohort

| **Patient identifier** | **Age at diagnosis** | **Histopathological diagnosis (if block detail differs)** | **Overall Grade (if selected block grade differs)** | **FIGO stage** | **Endometriosis** | **Primary specimen** | **Specimen site** |  |
| --- | --- | --- | --- | --- | --- | --- | --- | --- |
| 3705-0442 | 72 | CS | 3 | IIC | No | Yes | Ovary (L) |  |
| 3705-0449 | 71 | CS (carcinomatous element) | 3 | IIIC | No | No | PM (R) |  |
| 3705-0456 | 64 | CS | 3 | IIIC | No | Yes | Ovary (L) |  |
| 3705-0470 | 62 | CS (carcinomatous element) | 3 | IIIB | No | Yes | Ovary (ns) |  |
| 3705-0484 | 61 | CS (carcinomatous element) | 3 | IIIC | No | Yes | Ovary (R) |  |
| 3705-0500 | 70 | CS | 3 | IIC | No | Yes | PM (ns) |  |
| 3705-0510 | 58 | CS | 3 | IV | No | Yes | Ovary (L) |  |
| 666179 | 73 | ECC | 3 | IA | No | Yes | Uterus |  |
| 3705-0199 | 50 | EAE | 2 | IIIC | No | Yes | Uterus |  |
| 3705-0438 | 55 | EAE | 2 | IA | Yes | Yes | Uterus |  |
| 3705-0548 | 75 | EAE | 3 (2) | IIIA | No | Yes | Uterus |  |
| 3705-0553 | 69 | DCE (differentiated component) | 3 (1) | IV | No | Yes | Uterus |  |
| 3705-0142 | 60 | EAO | 2 | IA | No | Yes | Ovary (R) |  |
| 3705-0323 | 67 | EAO | 2 | IIA | Yes | Yes | Ovary (L) |  |
| 3705-0341 | 48 | EAO | 1 | IIA | No | Yes | Ovary (L) |  |
| 3705-0460 | 52 | EAO | 2 | IIB | Yes | Yes | Ovary (R) |  |
| 3705-0481 | 36 | EAO | 1 | IB | Yes | Yes | FT (R) |  |
| 3705-0482 | 59 | EAO | 2 | IC | Yes | Yes | Ovary (R) |  |
| 3705-0529 | 32 | EAO | 2 (1) | IB | No | Yes | Ovary (L) |  |
| 3705-0541 | 51 | EAO | 1 | IIA | No | Yes | Ovary (L) |  |
| 3705-0308 | 58 | MAO | 2 | IIB | No | Yes | Ovary (R) |  |
| 3705-0051 | 40 | MAOE | 2 | IIC | No | Yes | FT (L) |  |
| 3705-0145 | 37 | OCCC | 3 | IC | No | Yes | Ovary (L) |  |
| 3705-0207 | 50 | OCCC | 3 | IC | Yes | Yes | Ovary (R) |  |
| 3705-0346 | 57 | OCCC | 3 | IA | Yes | Yes | Ovary (R) |  |
| 3705-0379 | 48 | OCCC | 3 | IC | Yes | Yes | Ovary (L) |  |
| 3705-0383 | 45 | OCCC | 3 | IC | Yes | Yes | Ovary (R) |  |
| 3705-0416 | 66 | OCCC | 3 | IIB | Yes | Yes | Ovary (R) |  |
| 3705-0435* | 66 | OCCC | 3 | IV | NA | No | Lung (RLL) |  |
| 3705-0435 | 64 | OCCC | 3 | IIIA | No | Yes | PN (L) |  |
| 3705-0453 | 34 | OCCC | 3 | II (ns) | Yes | Yes | Ovary (R) |  |
| 3705-0464 | 54 | OCCC | 3 | IIA | No | Yes | Ovary (R) |  |
| 3705-0468 | 63 | OCCC | 3 | IIB | Yes | Yes | Ovary (L) |  |
| 3705-0497 | 61 | OCCC | 3 | IA | Yes | Yes | Ovary (L) |  |
| 3705-0514 | 55 | OCCC | 3 | IIB | No | Yes | Ovary (L) |  |
| 3705-0540 | 53 | OCCC | 3 | IIA | Yes | Yes | Ovary (Ns) |  |
| 3705-0544 | 48 | OCCC | 3 | IIIC | No | Yes | Ovary (Ns) |  |
| 3705-0545 | 62 | OCCC | 3 | IIIC | Yes | Yes | Ovary (R) |  |
| 3705-0558 | 53 | OCCC | 3 | IA | Yes | Yes | Ovary (R) |  |
| 3705-0487 | 68 | LGSOC | 1 | IIIB | No | No | PSW (L) |  |
| 3705-0493 | 36 | LGSOC | 1 | IIIA | No | Yes | Ovary (Ns) |  |
| 3705-0525 | 56 | LGSOC | 1 | IIIC | No | Yes | Ovary (L) |  |
| 3705-0475 | 76 | HGSOC | 3 | IIIC | No | Yes | FT (R) |  |
| 3705-0466 | 21 | SCCOHT | 3 | IV | No | Yes | Ovary (R) |  |
| 3705-0551 | 29 | SCCOHT | 3 | IIC | No | Yes | Ovary (R) |  |

Patients were aged between 21 and 76 years of age, and comprised of eight gynaecological subtypes, the most frequent being clear cell carcinoma of the ovary, *n* = 17.

CS, Carcinosarcoma; ECC, Endometrial Clear Cell Carcinoma; DCE, De-differentiated Carcinoma of the Endometrium; EAE, Endometrioid Adenocarcinoma of the Endometrium; EAO, Endometrioid Adenocarcinoma of the Ovary; MAO, Mesonephric Adenocarcinoma of the Ovary; MAOE, Mesonephric Adenocarcinoma of the Ovary and Endometrium; OCCC, Ovarian Clear Cell Carcinoma; LGSOC, Low-Grade Serous Ovarian Carcinoma; HGSOC, High-Grade Serous Ovarian Carcinoma; SCCOHT, Small Cell Carcinoma of the Ovary, Hypercalcaemic Type; PM, pelvic mass; PSW, pelvic side wall; R, right; L, left; ns, not specified; FT, fallopian tube; RLL, right lower lobe.
